# Recent insights on indirect mechanisms in developmental toxicity of nanomaterials

**DOI:** 10.1186/s12989-020-00359-x

**Published:** 2020-07-11

**Authors:** Battuja Batbajar Dugershaw, Leonie Aengenheister, Signe Schmidt Kjølner Hansen, Karin Sørig Hougaard, Tina Buerki-Thurnherr

**Affiliations:** 1 Laboratory for Particles-Biology Interactions, Empa, Swiss Federal Laboratories for Materials Science and Technology, Empa, Lerchenfeldstrasse 5, 9014 St.Gallen, Switzerland; 2 National Research Centre for the Working Environment, Copenhagen, Denmark; 3 Biotech Research and Innovation Centre, University of Copenhagen, Copenhagen, Denmark; 4 Department of Public Health, University of Copenhagen, Copenhagen, Denmark

**Keywords:** Nanomaterials, Developmental toxicity, Indirect toxicity pathways, Placental barrier, Pregnancy

## Abstract

**Background:**

Epidemiological and animal studies provide compelling indications that environmental and engineered nanomaterials (NMs) pose a risk for pregnancy, fetal development and offspring health later in life. Understanding the origin and mechanisms underlying NM-induced developmental toxicity will be a cornerstone in the protection of sensitive populations and the design of safe and sustainable nanotechnology applications.

**Main body:**

Direct toxicity originating from NMs crossing the placental barrier is frequently assumed to be the key pathway in developmental toxicity. However, placental transfer of particles is often highly limited, and evidence is growing that NMs can also indirectly interfere with fetal development. Here, we outline current knowledge on potential indirect mechanisms in developmental toxicity of NMs.

**Short conclusion:**

Until now, research on developmental toxicity has mainly focused on the biodistribution and placental translocation of NMs to the fetus to delineate underlying processes. Systematic research addressing NM impact on maternal and placental tissues as potential contributors to mechanistic pathways in developmental toxicity is only slowly gathering momentum. So far, maternal and placental oxidative stress and inflammation, activation of placental toll-like receptors (TLRs), impairment of placental growth and secretion of placental hormones, and vascular factors have been suggested to mediate indirect developmental toxicity of NMs. Therefore, NM effects on maternal and placental tissue function ought to be comprehensively evaluated in addition to placental transfer in the design of future studies of developmental toxicity and risk assessment of NM exposure during pregnancy.

## Background

Since the thalidomide scandal in the early 1960s, it has become evident that the placenta does not provide a tight barrier, and that fetuses are exceptionally susceptible to potentially toxic substances compared to adults, due to the phases of rapid growth, range of developmental events and often irreversible nature of the induced changes [1]. The first indications of developmental toxicity of nanosized particles came from epidemiological studies, showing association of particulate matter (PM) exposure with adverse pregnancy outcomes such as low birth weight, preterm birth and preeclampsia [2–4]. Recently, it has been confirmed that environmental black carbon reaches the fetal side of the placenta in exposed pregnant women [5]. With the advent of nanotechnology, novel NMs with unique properties can be industrially produced at large scales for application in food (reviewed in [6, 7]), cosmetics (reviewed in [7, 8]), medicine (reviewed in [9, 10]) and high-technology products (reviewed in [10, 11]). These engineered NMs further contribute to human exposure to nanosized particles, and due to their high reactivity, pose additional health risks. However, investigations of the toxicological effects of engineered NMs, especially in vulnerable populations such as pregnant women and their unborn children, have lagged behind the development of new applications. Importantly, to support safe-by-design and sustainable use of NMs, it is imperative to gain knowledge on the potential developmental toxicity of NMs and to understand the mechanisms underlying such toxicity.

**Fig. 1:**
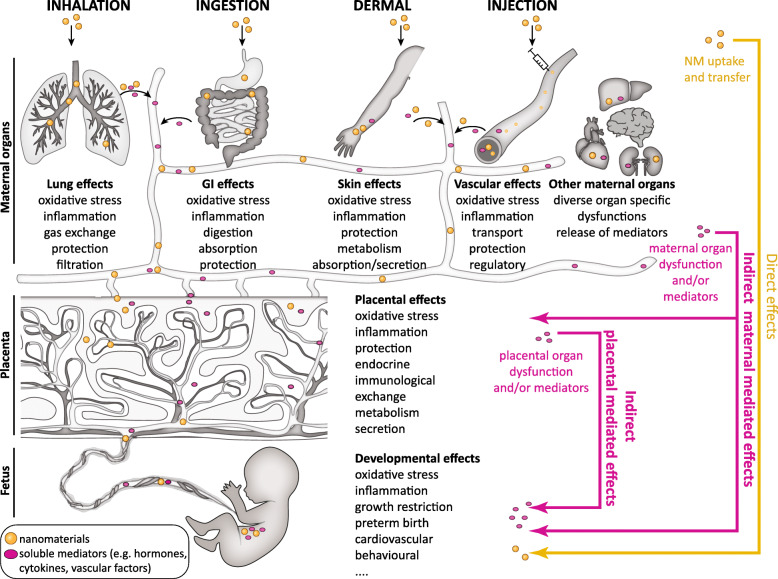
Scheme illustrating direct and indirect pathways of NM-mediated developmental toxicity

In principle, NMs can affect fetal development through two fundamentally different pathways: a direct and an indirect pathway [12] (Fig. 1), that, however, are not mutually exclusive. Direct developmental toxicity may arise from particles in maternal blood that cross the placental barrier [13–15] and directly damage fetal tissues due to their high surface reactivity and propensity to induce inflammation [16–18], reactive oxygen species (ROS) [19] and hence oxidative stress reactions [20–22], among others. Several FNMs are able to cross primary biological tissue barriers (e.g., lung [23, 24] and gastrointestinal (GI) tract [24, 25]) as well as the placenta [26–29], even if translocation is usually rather limited [30, 31]. Direct effects on embryonic and fetal tissues have been described for a variety of NMs in several in vitro studies as well as across species, including fish, chicken, and in vitro human stem cell (SC) models (reviewed in [32]). However, findings from organisms that lack a placenta or have a distinctly different placental structure might not directly correlate to the human condition.

The potential for NMs to affect fetal development by indirect pathways has been only marginally investigated and understood. Here, the concept is that NMs can interfere with fetal development in an indirect manner without being in direct contact with fetal tissue (Fig. 1). NMs deposited in primary maternal tissue barriers at the point of entry following oral, inhalation, dermal or intravenous (i.v.) exposure might induce oxidative stress and subsequently inflammation, leading to the release of inflammatory mediators and soluble signaling factors that can reach the placenta and fetus to induce potential toxic effects (maternal mediated developmental toxicity). Alternatively, particles reaching the placenta can cause similar responses in the placental tissue, compromising placental function and inducing the release of placental signaling factors, which might impair embryo-fetal development (placental mediated developmental toxicity).

The aim of this review is to (i) collect the current knowledge base on the indirect developmental toxicity of NMs, (ii) compile and describe already known signaling pathways, (iii) propose novel candidate pathways and (iv) suggest directions of future research needs.

### Risks and opportunities of NMs in pregnancy

For a proper risk assessment of NMs, a central aspect is to understand the exposure of pregnant women to NMs, including all relevant routes of exposure [33]. Due to the use of NMs in many consumer, high-technology and biomedical products, pregnant women could be exposed to NMs via inhalation, absorption through damaged skin, ingestion or injection (Fig. 1) (reviewed in [34, 35]). At production sites with applications of NMs, pregnant women can be exposed to NMs by inhalation, since the established protective legislation [36] does not come into action until the employer is made aware of the pregnancy, most often not until after the first 4–6 weeks. Even then, NM exposure might continue, as the regulation does not specifically regulate NM relative to pregnancy [37, 38]. Ingestion of NMs used as food additives, in food packaging material or personal care products, constitutes another realistic route of exposure during pregnancy. For example, the white food colorant E171 consists of particulate titanium dioxide (TiO_2_), with approximately 17–35% of the particles being within the nano-range (reviewed in [7, 39, 40]), and is present in toothpaste and various food products such as beverages, soups, cakes or candy in the European Union [41, 42]. In the United States, the dietary intake of TiO_2_ is estimated to be 1–2 mg/kg body weight per day for children, and 0.2–0.7 mg/kg body weight per day for other age groups [7, 42]. Dermal uptake of NMs present in personal care products, such as sunscreen, is expected to be minimal since the intact skin forms a tight barrier for NMs (reviewed in [43]). Finally, particles may be directly injected into the body in case of medical application of NMs (reviewed in [9, 44, 45]), but currently, nanomedical therapies during pregnancy are still in the investigational stage. For instance, King et al. demonstrated the potential of iRGD (9-amino acid cyclic peptide: CRGDKGPDC)-decorated liposomes loaded with insulin-like growth factor (IGF)-2 for the treatment of fetal growth restriction in mice [46]. An oxytocin receptor coated liposomal carrier loaded with the tocolytic drug indomethacin substantially decreased preterm birth rates in mice [47]. Nevertheless, before clinical use in pregnant women, not only the efficacy of the potential treatment in humans but also the safety of the NMs during pregnancy needs to be proven.

I.v. injection would make NMs readily systemically available. In contrast, only a low fraction of air and foodborne NMs would be expected to reach the systemic circulation and become bioavailable for maternal, placental and fetal tissues. Dermal exposure is expected to contribute very little to the systemic burden [27, 31]. Once NMs have reached the systemic circulation, they can distribute to maternal organs, including the placenta. As a highly perfused organ, the placenta is extensively exposed to circulating substances. Placental cells have been described to take up nanosized particles from the blood stream in experimental animals as well as the ex vivo human placenta perfusion model (e.g. [48–51]). Studies on placental translocation of NMs in rodents, in the human ex vivo and in in vitro placenta models have shown that some types of NMs are retained in the maternal circulation while others can pass the placenta (reviewed in [26, 52]). Placental transfer appears to partially correlate withphysicochemical properties of NMs, in particular particle size [26]. However, other factors such as the gestational stage or combined physico-chemical properties can also affect placental translocation of NMs, making this process difficult to predict [53]. As an example, a recent study demonstrated decreased fetal viability and growth, when 13 nm zinc oxide (ZnO) NPs were orally administered (7.2 mg/mouse) during organogenesis (gestational day (GD)7–16) in mice. However, when ZnO NP exposure occurred during the peri-implantation period (GD1-GD10) no fetal toxicity, but a slight change in placental weight, was observed [54].

For most routes of uptake (inhalation, ingestion and injection), gestational NM exposure has been associated with developmental toxicity for a variety of different NMs (extensively reviewed in [36, 55–58])*.* However, we have yet to identify the underlying mechanisms and which particle properties are of particular concern.

### Organ systems of relevance for pathways of indirect developmental toxicity

**Fig. 2:**
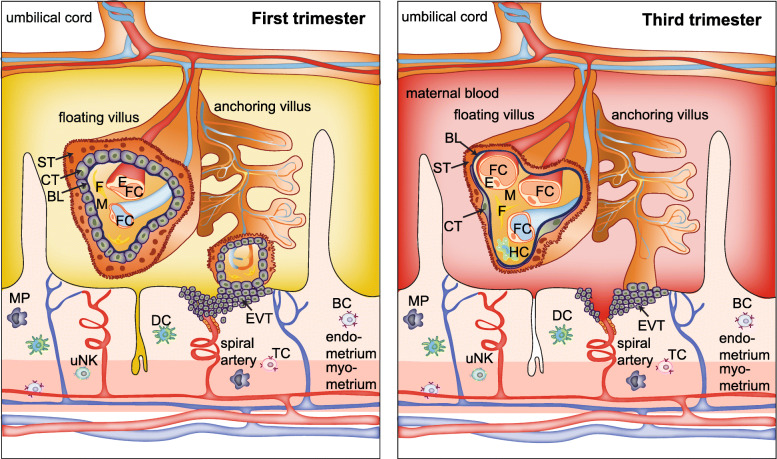
Scheme of the human placental barrier in early and late pregnancy. In the first trimester, the placental barrier consists of the syncytiotrophoblast (ST), cytotrophoblasts (CT), basal lamina (BL) and the endothelial cells (E) of the fetal capillaries (FC). Other cell types in the villous mesoderm include fibroblasts (F) and Hofbauer cells (HC). Various immune cells are also present in the maternal decidual tissue, including dendritic cells (DC), macrophages (MP), uterine natural killer cells (uNK), T cells (TC) and B cells (BC). Extravillous trophoblasts (EVT) of the anchoring villi invade the maternal spiral arteries (SA) and form a plug that prevents entry of maternal blood into the intervillous space, and uterine glands (UG) provide histiotrophic nutrition. After the first trimester, the EVT plug is released and placental villi are now surrounded by maternal blood. Towards the end of pregnancy, the placental barrier decreases in size by thinning of the ST layer and spreading of the CT layer, and the FCs move towards the periphery of the floating villi

For sure, the placenta should be a key focus in any mechanistic study on NM-mediated developmental toxicity due to its position at the interface between mother and fetus and its numerous essential functions during pregnancy. As a transient organ, the placenta starts forming after implantation of the conceptus in the uterine wall. It consists of tissues of maternal (decidua) and fetal origin (amnion, chorion) [59, 60]. Anatomically, the maternal side of the placenta comprises the multinuclear syncytiotrophoblast (ST) layer, which is supported by a basal membrane, underlying cytotrophoblast cells, mesenchymal tissue and the microvascular endothelium of the fetal small blood vessels (Fig. 2). This interface between the inner mucous membrane of the uterus (endometrium) and the fetus defines the degree to which maternally delivered substances reach the fetal tissue [61]. During pregnancy, the placenta undergoes dramatic structural and functional changes to fulfill the evolving needs of the developing fetus. During early pregnancy, the placental barrier is relatively thick (20–30 μm) and bilayered [62–64], but thins (2–4 μm) [65], becomes predominantly monolayered [62–64], and increases its surface area tremendously (to approx. 12 m^2^) towards the end of pregnancy to allow for efficient exchange of nutrients and gases required to sustain rapid fetal growth. Placental damage, disease or impairment of its development or function are responsible for numerous pregnancy complications, including preeclampsia [66], miscarriage [63, 67] and intrauterine growth restriction [63, 67], and can likely impact offspring health later in life [68]. It should also be highlighted that the placenta is the most species-specific organ among mammals and shows remarkable differences in global structure, tissue layer organization, trophoblast cell types [69, 70] as well as molecular features [71]. Therefore, translation from animal studies to the human situation should be done with caution, and the use of physiologically relevant placenta models is encouraged.

Also, maternal organs could mediate indirect developmental toxicity of NMs. Here, a focus should be on tissues at the port of entry that are in direct contact with particles such as the lung, the skin or the GI tract upon inhalation, dermal deposition or oral exposure, respectively. Uptake and accumulation of NMs in these tissues could affect organ functions locally, but effects may spread to distant sites, including the placenta or the developing fetus, if particles interfere with essential signaling pathways. This concept is nicely exemplified in a recent study in mice, where systemic adverse effects (i.e. increased retention of activated leukocytes, secondary thrombocytosis, and pro-inflammatory responses in secondary organs) were observed only upon inhalation exposure to carbon NPs, but not after intra-arterial injection of an equivalent dose of particles to bypass the lung [72]. The mechanism(s) underlying the observed indirect systemic toxicity of carbon NPs appeared to involve inflammatory responses of the lung tissue [72]. In addition to pro-inflammatory actions, NMs may also interfere with essential functions of the lung, skin or GI, such as gas exchange, digestion, nutrient uptake, metabolism or transport (Fig. 1). For instance, ZnO NPs can reduce iron and nutrient uptake and transfer at the intestinal barrier [73, 74].

### Evidence for indirect developmental toxicity

To gather an overview on potential indirect pathways in developmental toxicity, we searched the open literature for studies reporting adverse effects of NMs on gestation and fetal development in the absence of detectable materno-fetal particle transfer (Table 1). However, since direct and indirect toxicity pathways may jointly contribute to developmental toxicity, we also included studies that provide hints for indirect toxicity pathways even if placental transfer of NMs was detected (Table 2) or unknown (Table 3). The studies are briefly described below alongside the provided evidence and forwarded hypotheses for indirect mechanisms of toxicity.

**Table 1:** Studies with evidences for indirect fetotoxicity pathways without placental transfer of NMs

NP type/coating	NP size	exposure/model	application route/dose/exposure period	placental transfer	developmental toxicity (gestational and litter parameters)	developmental toxicity (other parameters)	hypothesis by authors on indirect toxicity pathways	publication
TiO_2_	5–6 nm	mouse	i.v./ 100 or 1000 μg/mouse/ GD9	not detected in fetus or placenta by ICP-MS	no overt fetal malformations or changes in pregnancy outcomes/ no impact on postnatal growth	behavioral deficits relevant to ASD and related neurodevelopmental disorders in neonates	maternal mediated unknown pathways due to absence of particles in placenta and fetal tissues	[75]
TiO_2_/CeO_2_	12.3 ± 0.1/ 22.4 ± 0.2 nm	mouse	instillation/ total 300 μg/mouse/ 100 μg at GD 2.5, GD 9.5 and GD 16.5)	Ti and Ce detected in the placenta but not in fetal tissues by ICP-MS	not evaluated	long-lasting impairment of lung development in offspring/ decreased placental efficiency together with the presence of NPs in the placenta/ no increase of inflammatory mediators in amniotic fluid, placenta or offspring lungs/ decreased pulmonary expression of VEGF-α and MMP-9 at the fetal stage (GD 17.5) and FGF-18 at the alveolarization stage (postnatal day 14.5)	probably involves placental insufficiency secondary to the presence of NPs in this organ with ensuing down regulation of critical mediators of lung development without any amniotic fluid or fetal lung inflammation/ not mediated via fetal or maternal lung inflammation	[76]
UV-Titan L181/polyalcohols	20.6 ± 0.3 nm	mouse	inhalation/ 1 h/day to 42 mg/m^3^/ GD 8–18	not detected in fetal liver by ICP-MS	no impact on gestational and litter parameters	moderate neurobehavioural deficits/ persistent lung inflammation in pregnant dams	dissolution and translocation of contaminating metal ions/ placental transfer of inflammatory cytokines released from NP-exposed maternal lung tissue	[77]
CuO	16 nm	mouse	inhalation/ 3.5 mg/m^3^ for 4 h/day/ GD 3–19	not detected by ICP-MS (similar Cu levels in placenta and fetus as controls)	survival rate of 7 week old pups reduced/ no impact on litter size, male/female ratio, body weight and lenght at birth	maternal pulmonary inflammation/ no histopathological changes of placenta tissue/ immunomodulatory effects in offspring (differential expression of several Th1/Th2 or other immune response genes in spleen)	changes in maternal inflammatory and immune responses	[78]
CdO	11–15 nm	mouse	inhalation/ 100 μg/m^3^ every other day or 230 μg/m^3^ daily for 2.5 h/ GD 4.5–16.5	Cd detected in placenta but not in fetus by gAAS and ICP-MS (Cd in placenta)	decreased incidence of pregnancy/ decreased fetal length/ delayed neonatal growth/ delayed maternal weight gain	altered placental weight	disruption in placental oxygen transfer by Cd [79]/ decrease in fetal length could be due to alterations in the fetal and/or maternal IGF system [80, 81]/ changes in the placental transport of zinc, vitamin B12, and other micronutrients due to placental Cd [82]	[83]
SWCNT /OH-functionalized	1–2 nm diameter and 5–30 μm length	mouse	oral/ 10 mg/kg or 100 mg/kg/ GD 9	not detected in placenta, fetal liver and fetal kidney by TEM	increased fetal resorption and fetal morphological and skeletal abnormalities at 10 mg/kg but not at higher dose	none	oxidative stress and inflammatory response in placenta/maternal tissue	[84]
SWCNT/non-oxidized, oxidized and ultra-oxidized	2.37 nm diameter, 0.85 μm length/ 1.58 nm diameter, 0.76 μm length/1.8 nm diameter, 0.37 μm length	mouse	i.v./ 10 ng to 30 μg/mouse/ GD 5.5	not detected by histological and micro-Raman analyses	high percentage of early miscarriages and fetal malformations; lowest effective dose 100 ng/mouse	vascular lesions and increased ROS in placenta/ increased ROS in malformed fetuses/ no increased ROS or evident morphological alterations in maternal tissues	oxidative stress in placental tissue	[85]
CoCr	29 nm	BeWo Transwell bilayer with underlying BJ fibroblasts	40 μg/ml/ 24 h	not detected by ICP-MS (similar Co and Cr levels in whole fetus as controls)	not applicable	DNA damage to the fibroblasts without significant cell death/ mechanism involving transmission of purine nucleotides (e.g. ATP) and intercellular signalling within the placental barrier through connexin gap junctions or hemichannels and pannexin channels	fetal damage mediated by placental tissue via release of mediators (e.g. ATP)	[86]
CoCr	29 nm	Bewo Transwell mono- or bilayers with underlying BJ fibroblasts or Oct4-hES	40 μg/ml / 24 h	not detected [86]	not applicable	DNA damage to fibroblasts or Oct4- hES cells only with BeWo double layer	indirect toxicity only across bilayered (human)/multilayered (mice) placental barrier	[87]
		mouse	i.v./ 0.12 mg or 0.012 mg/mouse/ GD 9.5 or 12.5	not detected by ICP-MS (similar Co and Cr levels in whole fetus as controls)	no pathological changes in neonatal visceral organ	DNA damage in neonatal blood and liver at GD 12.5 (placenta with three layers established) but not at GD 9.5 (nutrient exchange via uterus and yolk sac)/ no pathological changes in placenta		
CoCr	29 nm	Bewo Transwell bilayers and conditioned media transfer to NPC or NPC-derived astrocytes and neurons	40 μg/ml / 24 h	not detected [86]	not applicable	altered differentiation of human NPC and DNA damage in the derived neurons and astrocytes/ importance of autophagy and IL-6 release from placental tissue in NP-induced DNA-damaging singalling/ NPs can cause developmental neurotoxicity across placental barriers/ astrocytes are key mediators of this neurotoxicity/ fetal hippocampus is particularly affected in mice	exposure of the human placenta to CoCr NPs could initiate a singalling cascade that perturbs the relationship between astrocytes and neurons during neurodevelopment	[88]
		mouse	i.v./ 0.12 mg / dpc 9	not detected [87]	see [87]			

*ASD* autism spectrum disorders, *gAAS* graphite furnace atomic absorption spectroscopy, *FGF-18* fibroblast growth factor 18, *GD* gestation day, *ICP-OES* inductively coupled plasma optical emission spectrometry, *IGF* insulin growth factor, *i.v*. intravenous, *MMP-9* matrix metalloproteinase 9, *NP* nanoparticles, *NPC* neural progenitor cells, *ROS* reactive oxygen species, *TEM* transmission electron microscopy. *VEGF-α* vascular endothelial growth factor α

**Table 2:** Studies with evidences for indirect fetotoxicity pathways with placental transfer of NMs

NP type/coating	NP size	Exposure/model	application route/dose/exposure period	placental transfer	developmental toxicity (gestational and litter parameters)	developmental toxicity (other parameters)	hypothesis by authors on indirect toxicity pathways	publication
CdSe/CdS/ZnS quantum dots/PEG-phospholipid micelle	60 nm	mouse	i.v./ 100 mg/kg/ GD 17	increased Cd levels in umbilical cord and fetuses by ICP-MS	no gestational or fetal abnormalities or complications	no significant abnormalities in maternal blood biomarkers, histopathology or behavior	acute hepatocellular injury and possible stress caused by the injection did eventually contribute to the high miscarriage rate in macaques	[89]
		macaques	i.v./ 25 mg/kg/ GD 100	slightly increased Cd levels in fetal organs by ICP-MS	increased rate of miscarriage	no pathological changes in the placenta or major organs of the miscarried fetuses/ no inflammatory response or injury in maternal liver and lung tissues/ acute maternal hepatocellular injury		
Si and TiO_2_	70 nm and 35 nm	mouse	i.v./ 0.8 mg/mouse /GD 16 and 17	Si and TiO_2_ NP in placenta, fetal liver and brain by TEM	decrease of maternal body weight at GD 17/18/ lower uterine weights/ higher fetal resorption rates/ smaller fetuses	Si NP induced structural and functional abnormalities in placenta (decreased sFlt-1)/ heparin improved fetal weight and sFlt-1 levels in Si NP exposed mice	adverse effects are linked to structural and functional abnormalities in the placenta/ activation of coagulation, complement and oxidative stress in the placenta	[90]
Ag	12.3/ 22.4 / 10.4 nm	mouse	instillation/ total 300 μg/mouse/ 100 μg at GD 2.5, GD 9.5 and GD 16.5	Ag in placenta and fetal lung by ICP-MS	not evaluated	long-lasting impairment of lung development in offspring/ decreased placental efficiency together with the presence of NPs in the placenta/ no increase of inflammatory mediators in amniotic fluid, placenta or offspring lungs/ decreased pulmonary expression of VEGF-α and MMP-9 at the fetal stage (GD 17.5) and FGF-18 at the alveolarization stage (postnatal day 14.5)	probably involves placental insufficiency secondary to the presence of NPs in this organ with ensuing down regulation of critical mediators of lung development without any amniotic fluid or fetal lung inflammation/ not mediated via fetal or maternal lung inflammation/ combination of direct and indirect pathways possible due to low placental transfer of Ag	[76]
Ag	18–20 nm	mouse	inhalation/ 1 or 4 h/day to 640 μg/m^3^/ GD 0.5–14.5	Ag in maternal tissues, placenta and fetus by TEM/ no particles or ions detected by spICP-MS	increased number of resorbed foetuses	reduced oestrogen plasma levels (in 4 h/day exposures)/ increased expression of pregnancy-relevant inflammatory cytokines in the placentas/ no major pathological changes in the lung of the mothers and only minor lesions in maternal liver and kidney	adverse effects at least in part related to the release of inflammatory mediators by the placenta/ reduction of circulating oestrogen levels could indicate an endocrine disrupting action of Ag NPs	[91]
Ag/ PEGylate or carboxylate	2–15 or 5–15 nm	ex vivo human placenta perfusion	40 or 75 μg/ml / 6 h perfusion	low levels of Ag NPs > 25 nm in fetal circulation by spICP-MS	not applicable	low translocation of Ag ions and Ag NPs (below 0.02% of initial dose)/ considerable uptake of Ag NPs in placental tissue (4.2% of initial dose for AgCOONa; 0.75% for AgPEG)	low translocation but comparably high accumulation of ionic Ag and Ag NPs in placental tissue may result in indirect placenta-mediated developmental toxicity	[92]
Diesel exhaust	69 nm	rabbit	inhalation/ 1 mg/m^3^ for 2 h/day, 5 days/week/ GD 3–27	non-aggregated and “fingerprint” NP observed in maternal blood, trophoblasts and fetal blood by TEM	growth retardation	reduced placental efficiency/ reduced placental vascularization/ reduced plasma insulin and IGF1 concentrations/ in second generation, fetal metabolism was modified	adverse effects on placental structure and function and reduced plasma IGF-1 may contribute to the observed growth retardation/ effects could be due to either NP or contaminants (e.g. PAHs)	[93]
MWCNT/ oxidized and ^99m^Tc	1–2 μm length, diameter 20–30 nm	mouse	i.v./ 20 mg/kg/ GD17	NPs in placental tissue and foetal liver, lung and heart by radioactivity measurements	poor embryo development/ fetal growth restriction/ embryonic death/ abortion/ reduced fetal weight/ fetal heart and brain damage	decreased progesterone levels and increased oestradiol levels in serum/ decreased VEGF levels and increased ROS amounts in placental tissue/ number of placental blood vessels decreased	fetal growth restriction due to vascular reduction in the placenta/ toxicity higher in first time pregnancies as adaptations in the placenta may occur/ oMWCNT affect secretion of progestational hormones	[94]
SWCNT and MWCNT/amine-functionalized (PL-PEG-NH_2_)/ ^64^Cu for translocation	SWCNT:1–2 nm diameterMWCNT: < 8 nm, 20–30 nm or 50 nm diameter, 500–2000 nm length	mouse (p53+/+; p53 +/−; p53 −/−)	i.v./ 2 mg/kg or 5 mg/kg/ GD 10.5, 12.5 or 15.5/ single or repeated doses	all CNTs in placental tissue and fetal liver by positron emission tomography	larger sized MWCNT restricted the development of fetuses and induced brain deformity (only at GD 10.5 and only in p53−/− fetuses)/ SWCNTs and smaller sized MWCNTs showed no or less fetotoxicity	MWCNTs directly triggered p53-dependent apoptosis and cell cycle arrest in response to DNA damage/ N-acetylcysteine (antioxidant) pevented CNT-induced nuclear DNA damage andreduce brain development abnormalities	placenta mediated toxicity thorugh interference with placental function	[95]

*FGF-18* fibroblast growth factor 18, *GD* gestation day, *ICP-OES/MS* inductively coupled plasma optical emission spectrometry/mass spectroscopy, *IGF* insulin growth factor, *i.v.* intravenous, *MMP-9* matrix metalloproteinase 9, *NP* nanoparticles, *PAH* polycyclic aromatic hydrocarbons, *ROS* reactive oxygen species, *spICP-MS* single particle ICP-MS, *TEM* transmission electron microscopy; *VEGF-α* vascular endothelial growth factor α

**Table 3:** Studies with evidences for indirect fetotoxicity pathways with unknown placental transfer of NMs

NP type/coating	NP size	Exposure/model	application route/dose/exposure period	developmental toxicity (gestational and litter parameters)	developmental toxicity (other parameters)	hypothesis by authors on indirect toxicity pathways	publication
TiO_2_	21 nm	rat	inhalation/ cummulative lung burden of 525 μg/ GD 11–16	not evaluated	increased placental vascular resistance and impaired umbilical vascular reactivity	impaired fetoplacental vascular reactivity/ altered placental reactivity and anatomy	[96]
Si	70 nm	mouse	i.v. injection/ 0.025 or 0.04 mg/g/ GD 13–14	increased fetal resorption and reduced fetal weight at 0.04 mg/ml	particle uptake in placenta/ 0.04 mg/ml: abnormalities in placental structure and reduced placental weight/ nanosilica upregulated the inflammasome component NLRP3 and induced placental inflammation and ROS, resulting in pregnancy complications/ pregnancy complications were dependent on the balance between an inflammatory cytokine (IL-1a) and an anti-inflammatory cytokine (IL-10)/ complications were completely prevented by either inhibition of ROS generation or forced expression of IL-10	placental inflammation	[16]
CdTe quantum dots	2 nm	rat	i.p./ 5, 10 or 20 mg/kg/ GD 13	dose dependent embryotoxicity/ reduced survival rate of fetuses/ reduction of fetal body length and mass/ disturbed ossification of limbs	placental tissue damage (decreased placental weight, abnormal morphological features)	impeded embryogenesis due to the placental damage rather than QD penetration and accumulation in the fetuses/ distinct developmental toxicity effects than upon Cd^2+^ exposure	[97]
CdTe quantum dots/ CuO	3 nm/ 10–20 nm	BeWo/HVMF placental microtissues	0–25 μg/mL/ 24 h	not applicable	reduction of β-hCG secretion at sub-lethal concentrations	interference with hormone release	[98]
Dendritic polyglycerol/sulfate, amine or neutral	5–7 nm	first trimester placental explants	10 nM and 1 μM/ 24 h	not applicable	charge-dependent accumulation of particles/ no major acute toxicity but reduced secretion of β-hCG for charged particles at the lower concentration	potentially hazardous influences of charged dendritic polygylcerol particles on early placental physiology by reduction of β-hCG hormone levels	[99]
MWCNT	13 μm length	mouse	i.p or intratracheally/ 2,3,4 or 5 mg/kg/ GD 9	fetal malformations/ increased leucocyte and related hemocyte number and increased weight of spleen in dams	none	inflammatory mechanism	[100]
CB	14 nm	mouse	inhalation: 42 mg/m^3^/ 1 h/day/ GD 8–18 instillation: 2.75, 13.5 or 67 μg/mouse/ GD 7, 10, 15 and 18	neither inhalation nor instillation affected gestation and lactation	DNA strand breaks in maternal and offspring liver after inhalation but not instillation exposure/ persistent lung inflammation in exposed mothers	translocation across lung, GI tract and placenta expected to be very low for highly insoluble CB; changes in signalling cascades proposed e.g. inflammatory molecules	[17]
CB	14 nm	mouse	intratracheal instillation/ 2.75, 13.5 or 67 μg/mouse/ GD 7, 10, 15 and 18	see (Jackson 2011)	changes in the expression of several genes and proteins associated with inflammation in maternal lungs/ hepatic response in offspring at highest dose	responses in newborns secondary to inflammation in dams	[101]
CB/ TiO_2_/ DEP	not determined	mouse	intratracheal instillation/ 50 μg/mouse/ GD 14	not evaluated	increased allergic susceptibility in offspring	components of DEP (especially PAHs) could mediate pro-allergic effects by increased production of Th2 cytokines (e.g., IL- 4), known to be important mediators of allergy and asthma	[102]
graphene oxide	4 different sizes (1–40 μm; 20 nm-1.4 μm; 0.2–1 μm; 10–30 μm)	2D BeWo or BeWo Transwell cultures	0–40 μg/mL/ 6 h, 24 h or 48 h	not applicable	particle uptake in BeWo cells/ no major acute toxicity but reduced secretion of β-hCG and transient reduction in barrier integrity	interference with hormone release and barrier integrity	[103]
PM_2.5_	< 2.5 μm	human	ambient PM_2.5_ exposures over the entire pregnancy from 5.54 to 29 μg/m^3^	not evaluated	positive relationship between PM2.5 exposure during preconception and pregnancy and intrauterine inflammation	intrauterine inflammation upon PM2.5 exposure in pregnancy may influence subsequent fetal growth, development,and health outcomes	[49]
PM_10_	< 10 μm	human	mean exposure levels during pregnancy were 30.3 μg/m^3^ for PM_10_ and 39.9 μg/m^3^ for NO_2_	not evaluated	short-term maternal PM10 exposure was modestly associated with elevated maternal CRP levels in early pregnancy and that long-term maternal PM10 and NO_2_ exposure during pregnancy was associated with elevated fetal CRP levels at delivery	exposure to air pollution during pregnancy may lead to maternal and fetal inflammatory responses	[104]
PM_10_	< 10 μm	human	mean exposure levels during pregnancy were 30.3 μg/m^3^ for PM_10_ and 39.9 μg/m^3^ for NO_2_	not evaluated	associations of PM10 and NO_2_ exposure with changes in fetal sFlt-1 and PlGF levels at delivery/ higher PM10 and NO_2_ exposures were associated with lower placenta weight/ air pollution exposure was not consistently associated with other markers of placental growth and function	maternal air pollution exposure may influence markers of placental growth and function	[104]

*BC* black carbon, *CB* carbon black, *DEP* diesel exhaust particles, *GD* gestation day, *ICP-OES* inductively coupled plasma optical emission spectrometry, *hCG* human chorionic gonadotropin, *HVMF* human villous mesencyhmal fibroblasts, *IGF* insulin growth factor, *i.p*. intraperitoneally, *i.v*: intravenous, *NP* nanoparticles, *PAHs* polycyclic aromatic hydrocarbons, *PM* particulate matter, *ROS* reactive oxygen species, *TEM* transmission electron microscopy

#### Studies without detectable placental particle transfer

We identified a total of ten studies that reported developmental toxicity in the absence of detectable NM translocation across the placental barrier (Table 1). Most used pregnant mice as the experimental model, but a few studies used in vitro cell culture systems for more mechanistic studies. Gestational and litter parameters were affected in four of the murine studies, including reduced survival rate of offspring from dams inhaling copper oxide (CuO) (3.5 mg/m^3^ at GD 3–19) [78], decreased fetal size and delayed neonatal growth from cadmium oxide (CdO) NP inhalation (100 μg/m^3^ or 230 μg/m^3^ at GD 4.5–16.5) [83], and increased fetal resorption and malformations following maternal exposure to SWCNTs by the oral (10 or 100 mg/kg body weight at GD 9) [84] and i.v. route (10 ng to 30 μg/mouse at GD 5.5) [85]. Other studies described effects on placental structure and function, offspring lung development and function and neurodevelopment. Regarding the placenta, intratracheal instillation of TiO_2_ and cerium dioxide (CeO_2_) NPs (total 300 μg/mouse: 100 μg on GD 2.5, 9.5 and 16.5, respectively) decreased placental efficiency [76], injection of CdO NPs altered placental weight [83] and injection of SWCNTs induced vascular lesions and increased placental level of ROS [85]. Gestational NM exposure can also affect maternal and fetal lungs as exemplified by maternal lung inflammation induced by inhalation of TiO_2_ (42 mg/m^3^ on GD 8–18) [77] or CuO NPs (3.5 mg/m^3^ on GD 3–19) [78], or long-lasting impairment of lung development in the offspring resulting from maternal intratracheal instillation of TiO_2_ or CeO_2_ NP [76]. Neurodevelopmental abnormalities, like reactive astrogliosis and increased DNA damage in the fetal hippocampus, have been observed after injection of cobalt-chrome (CoCr) NPs into pregnant mice on GD 9 (0.12 mg per mouse) [88]. In a similar direction, both maternal inhalation with 42 mg/m^3^ (GD 8–18) [77] and injection with 1000 μg/mouse (GD 9) [75] of TiO_2_ NPs caused behavioral deficits in the offspring.. Finally, immunomodulatory effects were reported upon CuO inhalation [78]. Importantly, NM translocation to the fetus was addressed but not observed in these studies, therefore strongly supporting the presence of indirect toxicity pathways. However, it is conceivable that a small amount of particles might have crossed the placental barrier, which were below the detection limit of the applied analytical techniques (i.e. ICP-MS, gAAS, TEM, histological and micro-Raman analysis), as for example suggested by Hougaard et al., 2010 [77]. Moreover, for soluble NPs (e.g. CuO), placental translocation of small quantities of dissolved ions might also partially account for developmental toxicity even in the absence of particle transfer. Nevertheless, the adverse effects upon CuO inhalation in mice observed by Adamcakova-Dodd et al. [78] were not associated with detectable increase in fetal or maternal blood Cu levels. Proposed pathways for indirect developmental toxicity included both placenta- and maternally mediated secondary mechanisms. Maternally mediated pathways comprised oxidative stress, inflammatory, immune and endocrine responses [75, 77, 78, 83, 84], whereas placental mediated pathways involved oxidative stress, inflammation, placental insufficiency, release of mediators (e.g., ATP, IL-6) and changes in placental transport of zinc, vitamin B12, micronutrients or oxygen [76, 79–88]

#### Studies with placental particle transfer

Several publications suggested a role for indirect developmental toxicity of NMs even if particles in some cases were shown to cross the placental barrier and adverse effects could have resulted from direct embryo-fetal exposure (Table 2). The gestational and litter parameters described in these studies include increased rate of miscarriage from quantum dot (QD) injection [89], smaller fetuses and increased fetal resorption from silica dioxide (SiO_2_) and TiO_2_ NP injection [90], growth retardation from diesel exhaust particle (DEP) inhalation [93] or multi-walled carbon nanotube (MWCNT) injection [94] and fetal organ damage from QD [89] or SWCNT/MWCNT injection [94, 95]. The maternal parameters reported were decreased maternal body weight upon SiO_2_ and TiO_2_ NP injection (0.8 mg/mouse on GD 16 and 17) [90] and hepatocellular injury from QD injection (100 mg/kg body weight on GD 17 in mice and 25 mg/kg on GD 100 in monkeys) [89]. Paul et al. observed long-lasting impairment of lung development in offspring of pregnant mice intratracheally instilled with silver (Ag) NPs (total 300 μg/mouse: 100 μg on GD 2.5, 9.5 and 16.5, respectively) and suggested that the underlying mechanisms may involve placental insufficiency with ensuing down-regulation of critical mediators of lung development [76]. Other proposed placenta mediated indirect pathways involve adverse effects of NMs on placental structure and function [90, 92, 93, 95], the release of placental inflammatory mediators [91], reduction in placental vasculature [93, 94] and activation of coagulation, complement and oxidative stress in the placenta [90] and disruption of endocrine signaling [91, 93].

#### Studies with unknown placental particle transfer

In several studies, placental translocation was not assessed, but the authors nevertheless postulated a role for indirect pathways of developmental toxicity based on observed interference of NMs with maternal organs or placental function (Table 3). Most of these studies did not evaluate gestational and litter parameters, but alterations of these parameters have previously been described following maternal exposure to TiO_2_ NPs [90], PM [2–4] and DEPs [93]. Injection of SiO_2_ NPs in pregnant mice (0.025 or 0.04 mg/g body weight on GD 13 and 14) resulted in increased fetal resorption and reduced fetal weight, possibly through particle-induced inflammatory responses in the placental tissue [16]. These complications were entirely prevented by ROS inhibitors or forced expression of IL-10 [16]. Maternal or intrauterine inflammatory pathways were also proposed to mediate developmental toxicity from exposure to air pollution [49, 104] carbon black (CB) [17, 101] and MWCNTs [100]. Besides inflammatory pathways, interference with the placenta (structure, growth or function/reactivity) has been suggested to constitute another indirect pathway for developmental toxicity of air pollution particles [104], cadmium telluride (CdTe) QDs [97], TiO_2_ NP [96] in vivo or graphene oxide (GO) in vitro [103]. For prenatal exposure to CB, TiO_2_ and CeO NPs (intratracheal instillation: 50 μg/mouse on GD 14), Fedulov et al. observed increased allergic susceptibility in the offspring that was proposed to occur due to NM-induced production of Th2 cytokines in maternal lungs [102].

Overall, for all of the three study categories (studies investigating but not detecting placental transfer (Table 1), studies detecting placental transfer (Table 2) and studies with unknown placental transfer (Table 3)), indications of potential indirect toxicity pathways mediated by maternal and/or placental tissue have been identified. Moreover, considering that maternal and placental tissues are probably exposed to NMs at earlier time points and higher dose levels compared to the fetal compartment, extending the focus from direct to indirect effects is of key importance to advance our understanding of risks associated with NM exposure during pregnancy.

### Candidate pathways for indirect developmental toxicity

Developmental toxicity is mostly assessed in experimental animals and often centers on classical gestational and litter parameters. In light of the growing evidence for maternal and placenta mediated developmental toxicity of NMs, it is crucial to perform more comprehensive assessments of placental, maternal and fetal/offspring tissue and organ functions. In this section, we will compile and discuss the different indirect pathways forwarded in the reviewed literature, to outline how NMs may adversely affect developmental outcomes without direct exposure of the fetus to NMs. Although we will mostly describe individual indirect pathways, these are likely interlinked and jointly contribute to adverse fetal outcomes, potentially even in conjunction with direct toxicity pathways.

#### NM-induced oxidative stress and inflammatory responses

The placenta has a very high turnover of oxygen and ROS are generated continuously, with the main source being the mitochondrial respiratory chain. Overall, the balance between oxidants and antioxidants is vital for maintaining physiological homeostasis. During normal pregnancy conditions, ROS are eliminated by the corresponding and abundant production of antioxidants by the feto-placental unit. If this redox balance is disturbed pathological conditions may emerge [105]. Several types of NMs induce the generation of ROS, either directly or via activation of oxidative enzymatic pathways [106–109]. Excessive amounts of ROS may overwhelm the capacity of the intrinsic antioxidants and result in a condition of oxidative stress [110]. ROS can damage cells by interaction with lipids, proteins and DNA and by induction of inflammation [108, 111]. Placental inflammation is a well-established risk factor for pregnancy and fetal development [112]. If NMs are taken up by placental cells, the subsequent generation of ROS, oxidative stress and inflammation has been hypothesized to represent one indirect mechanistic pathway by which NMs can interfere with placental development and function, and hence, with fetal development [36, 55].

Inhaled particles that deposit in the lung alveoli can also locally induce ROS and inflammation. This will often be accompanied by increased transcription of pro-inflammatory genes and ultimately the production of inflammatory mediators, such as cytokines and acute-phase proteins that can become systemically available [113, 114]. It is increasingly being described that maternal inflammation is a potent modulator of fetal development and that the developing nervous system may be especially sensitive. Maternal inflammation has been proposed to constitute an immune challenge to the fetus that could prime early alterations in the inflammatory response systems and, in turn, disrupt development and maturation of the central nervous system and enhance sensitivity to additional stress factors [115]. Maternal inflammation may not necessarily result in fetal inflammation, but the placenta may act as a sensor of maternal inflammation and subsequently adapt to the inflammatory environment and may act both as a target and a producer of inflammatory mediators [116].

Shirasuna et al. (2015) elegantly aimed to investigate if NPs induce pregnancy complications through placental inflammation [16]. Pregnant mice were injected i.v. with 0.04 mg/kg body weight of 70 nm silica particles on GD 13 and 14. This exposure increased fetal resorptions, induced placental dysfunction, ROS generation and infiltration with neutrophil granulocytes (3-fold). Also, placental protein levels of several inflammatory cytokines were significantly increased (IL-1β, IL-6, TNF-α, and CCL2). Pre-treatment with the antioxidant N-acetyl cysteine (NAC) almost completely reversed the placental and fetal effects of the injected NPs, reduced placental ROS levels, cell infiltration and secretion of IL-1β and IL-1α. Findings in specific knock-out mouse strains indicated that the balance between the inflammatory cytokine IL-1 and the anti-inflammatory IL-10 was pivotal for induction of adverse effects. Therefore, the study was repeated with forced expression of IL-10 by injection of adeno-associated virus vectors encoding murine IL-10. Again, the placental and fetal effects of the injected NPs were reversed. Of note, inhibition of placental phagocytosis and hence uptake of NPs significantly blocked IL-1β and IL-1α secretion, indicating that uptake of NPs into the cells might has been involved in inducing inflammatory pathways in placental cells [16].

The induction of placental ROS by NMs was also addressed by Qi et al. (2014) [94]. Pregnant mice were injected i.v. with 20 mg of oxidized (o-) MWCNTs/kg on GD 17. Exposure increased the ROS levels in placentas, but not in maternal plasma, indicating that the placenta may respond more vigorously or faster to o-MWCNTs than other maternal tissues. The observation of placental implication in developmental toxicity has some resemblance to reports on the effects of SWCNTs, TiO_2_ and silica NPs [85, 90].

Also, other studies have attempted to investigate the degree to which oxidative stress contributes to developmental effects by administering antioxidants alongside the maternal exposure to NMs [55]. Onoda and co-workers investigated the protective effects of antioxidants on the development of reactive astrogliosis in the offspring that had been observed following maternal intranasal instillation of CB NM (95 μg/kg body weight) on GD 5 and 9 in several previous studies. N-acetyl cysteine or ascorbic acid were administered intraperitoneally to pregnant mice prior to CB instillation. N-acetyl cysteine partly prevented, whereas ascorbic acid slightly enhanced, astrogliosis in the offspring [117]. Another study investigated the developmental effects of MWCNTs injected intravenously to pregnant p53+/− mice (2 mg/kg or 5 mg/kg body weight on GD 10.5, 12.5 or 15.5 as a single or repeated dosis). MWCNTs increased the incidence of brain defects in the offspring and decreased offspring survival rate after birth. The underlying mechanism seemed to involve MWCNTs directly triggering p53-dependent apoptosis and cell cycle arrest in response to DNA damage. Co-injection of an antioxidant markedly decreased the number of fetuses with brain defects, indicating that oxidative stress may be implicated. In this study, MWCNTs were found to distribute to the placenta and fetal liver but were not observed in the fetal brain [95]. Finally, intratracheal instillation of 4–5 mg MWCNTs/kg to pregnant mice on GD 9 was found to induce fetal malformations and to significantly increase maternal leukocyte counts in peripheral blood. At a lower dose of 3 mg/kg, no abnormality occurred. This suggests that maternal inflammation may be contributing to fetal toxicity [100].

Overall, these findings offer evidence of the involvement of oxidative stress in developmental toxicity of NMs. It is, however, important to keep in mind that observation of protection by antioxidants does not specify whether the effects occurred due to oxidative stress-induced directly by particles or indirectly via other mechanistic pathways. In some studies, particle exposure also induced pregnancy complications, such as fetal death, that could be associated with apoptosis and hence generation of increased levels of ROS. Therefore, it is not possible to deduct whether the increases in ROS levels occurred due to particle exposure or pregnancy complications.

#### NM interference with placental toll-like receptors

Several cell types express receptors for recognition of pathogen-associated molecular patterns present on the surface of microorganisms. Probably the best-described group of pattern recognition receptors are the TLRs, a group of evolutionary conserved transmembrane proteins [118]. Until now, 11 mammalian TLRs have been defined. TLR 4 is crucial for response to lipopolysaccharide (LPS) and, thereby, to gram-negative bacteria. TLR 2 recognizes a broader array of molecular patterns from bacteria and fungi. Ligand recognition by the TLRs mostly results in the activation of the intracellular signaling pathway of NFκB, ultimately increasing the production of cytokines and antimicrobial factors [119].

The human placenta expresses all of the TLRs, varying in a temporal and spatial manner [120]. Activation of trophoblast TLRs enhances cytokine expression, which may be followed by significant recruitment of immune cells (macrophages, NK cells) to the placenta. TLR-activation is associated with negative pregnancy outcomes (preterm labor, fetal loss and preeclampsia), but also plays a role in long-term adverse outcomes in the offspring, such as the function of the immune and central nervous systems [119]. Placental TLRs may, however, also be involved in the protective effects hypothesized to occur in case of “adequate” non-infectious microbial exposure as proposed by the hygiene hypothesis [121].

Accumulating evidence indicate that TLRs might recognize some NMs and activate similar pathways as upon contact with LPS and bacteria [122, 123]. Hence, MWCNTs have been shown to induce DNA damage in human lung epithelial cells due to the activation of TLR 9 and subsequent generation of nitric oxide (NO) [124]. Also, SWCNTs have been reported to provoke chemokine secretion in macrophages via the TLR 2/4-MyD88-NFκB signaling pathway [125]. Interestingly, when graphene oxide was tested in the same setup, no such response was elicited, indicating that TLRs may have a differential preference for subgroups of NMs [125]. In silico investigations show that the internal hydrophobic pockets of TLR 4 might be able to bind small-sized carbon nanostructures such as fullerenes and CNTs [126]. TLR 4 has, however, been shown to also recognize non-carbonaceous NMs, such as iron and TiO_2_ NPs, to promote inflammatory responses [127–129].

In the human placenta, TLR 2 and TLR 4 have been observed to lack in the ST but to be expressed in villous and extravillous trophoblasts, at least during early pregnancy [119]. This could indicate that, at this stage, the placenta responds primarily to pathogen-associated molecular patterns if the ligand has broken through the outer layer [119]. Therefore, NMs would need to be internalized by the trophoblast for TLR activation. Interestingly, several studies in the ex vivo human placenta model and experimental animals report that nanosized particles accumulate in placental tissue [130–132] and that particles can be visualized in trophoblasts [90, 91, 133–135].

Activation of placental TLRs by NMs would implicate the presence of NMs in maternal blood and their uptake/penetration into the ST. Some TLRs do also respond to endogenous molecules via so-called danger-associated molecular patterns, including, but not restricted to, ROS and proteins released from dead or dying cells [119]. Hence induction of ROS or inflammation by NMs in placental tissue, via direct or indirect pathways, may indirectly activate TLRs.

#### NM interference with endocrine signaling

Endocrine signaling pathways are central in mediating physiological and metabolic adaptations required for a successful pregnancy and are orchestrated by the placenta and the maternal endocrine organs (e.g., the pituitary, thyroid and adrenal glands, and the ovaries) [136, 137]. First evidence that NMs can have endocrine-disrupting activity came from studies in non-pregnant individuals, where NMs have been reported to affect levels of both female and male sex hormones in vitro and in vivo (reviewed in [138, 139]). For example, exposure of female and male rats to nickel (Ni) NPs by gavage resulted in altered hormone regulation (FSH and LH levels were elevated and estradiol lowered in females while testosterone and FSH levels were diminished in males) and induced pathological changes in testes and ovaries (reviewed in [140]). However, it largely remains to be established if NMs might act as endocrine disruptors during pregnancy and how this could potentially affect pregnancy and offspring health later in life.

In pregnancy, one of the critical hormones secreted by the human placenta is human chorionic gonadotropin (hCG) [137]. It supports the function of the corpus luteum, a transient ovarian structure particularly important in the early gestational phase, which secretes ovarian progesterone and estrogens to maintain a successful pregnancy [141]. hCG also regulates the formation of the ST [142, 143], modulates immune responses [143], ensures uterine quiescence [143], promotes angiogenesis of the endometrial spiral arteries [143, 144], and dilates these vessels to enhance maternal blood flow [145]. Due to these various crucial functions of hCG, disturbances in the tightly regulated levels of this hormone could, therefore, increase the risk of adverse pregnancy outcomes [146]. A few in vitro studies using BeWo trophoblast monocultures [103], 3D placental co-culture microtissues (BeWo cells/primary human villous mesenchymal fibroblasts) [98] or first trimester human placental explants [99] showed a significant reduction of hCG release after exposure to GO, CdTe and CuO NPs or dendritic polyglycerol NPs, respectively. This emphasizes that disturbances in hCG release should be considered in developmental toxicity studies.

Also, the steroid hormones estrogen (reviewed in [147]) and progesterone (reviewed in [148]) are indispensable to maintain human pregnancy. Estrogens are essential for vasodilation and local angiogenesis due to their close interaction with angiogenic factors like vascular endothelial growth factor (VEGF) and placental growth factor (PLGF) (reviewed in [147]). Dysregulation of estrogen secretion could, therefore, play a major role in the development of preeclampsia and other adverse conditions during pregnancy. Progesterone is essential for the reproductive process. Altered progesterone secretion has been associated with miscarriage and preterm birth [148]. So far, only a few descriptive studies reporting NM effects on steroid hormone levels in pregnant animals are available. Inhalation of Ag NPs decreased estrogen plasma levels in pregnant mice, but it was unclear if the Ag NP exposure caused the increase in observed fetal resorptions [91]. In another study, serum levels of progesterone decreased while estradiol levels increased in pregnant mice injected with MWCNTs [94]. Furthermore, VEGF levels and placental vascularization decreased and were linked with the reported growth restriction in the offspring [94], indicating a potential interconnection between endocrine and vascular pathways.

Other relevant placental and maternal hormones include human placental lactogen, the placental growth factor prolactin, the neuropeptides serotonin, melatonin and oxytocin (reviewed in [136, 137, 141, 149, 150] as well as IGFs (reviewed in [150–153]). Inhalation of DEPs in pregnant rabbits resulted in intra-uterine fetal growth retardation that was accompanied by reduced placental efficiency alongside a decrease in fetal plasma levels of IGF-1 [93], a peptide hormone essential for the regulation of feto-placental growth (reviewed in [150, 153]). However, it remains to be shown in how far the observed changes in fetal IGF-1 levels are responsible for the observed developmental toxicity. Moreover, further clarification is needed if contaminants (e.g. metals or polyaromatic hydrocarbons) associated with the particles could be involved or even be responsible for the adverse effects of DEPs on fetal development.

In summary, the few available studies indicate that NMs might act as endocrine disruptors and interfere with hormonal signaling in pregnancy. The underlying mechanisms are not yet well understood and might include direct effects on hormone biosynthesis and secretion, interference of NMs with hormone-receptor binding on target cells or with downstream signaling pathways (reviewed in [140]) as well as indirect effects, e.g., via the induction of inflammatory processes, which have been shown to cause endocrine imbalance [154]). Overall, a better understanding of the possible interference of NMs with the endocrine system during pregnancy is still needed and should include long-term studies since endocrine responses often take time before NM-triggered effects are manifested.

#### NM interference with vascular signaling and utero-placental development and function

To accommodate the developing fetus, extensive vascular adaptations take place in the uterus and placenta. Maternal blood volume increases around 45% [155] and uterine placental blood flow increases ten-fold [156]. Maternal blood pressure usually decreases or remains unchanged during pregnancy. This is mainly achieved through a decreased uterine vascular resistance, which is ultimately determined by a combination of an increase in vessel diameter, a reduced vascular tone (vasodilation) and an establishment of the placenta [156]. To ensure that vascular remodeling does neither harm the mother nor the fetus, microcirculatory regulation of blood flow is crucial [157, 158]. Early in pregnancy, the uterine spiral arteries are structurally converted from small diameter arteries into low-resistance large diameter vessels by interaction with the fetal placental extravillous trophoblasts that invade the myometrium and the spiral arteries [159]. Some chemicals have been shown to interfere with vascular remodeling and development of the placenta, thereby impairing oxygen and nutrient delivery to the fetus and ultimately increasing the risk for adverse pregnancy outcomes (reviewed in [160, 161]). Several epidemiological studies have found that components of air pollution, including PM with 2.5 μm or less in diameter (PM_2.5_)_,_ were associated with increased risk of pregnancy-induced hypertensive disorders [2].

For engineered NMs, studies in experimental animals indicate that maternal NM exposure during gestation interferes with maternal vascular reactivity and placental vascular development and function. Exposure to NMs has been associated with increased tone and contractility of the uterine vasculature [162–166]. In some studies, placentas from exposed pregnant rats were larger, while their offspring were smaller compared to the control group, indicative of effects downstream from the uterine vascular impairment [163, 166]. As an example, i.v. administration of MWCNTs (100 μg/kg body weight) or Ag NPs (200 μg/rat) to pregnant rats at GD 17–19 increased the contractility of the uterine artery. The maternal mesenteric artery and thoracic aorta were unaffected, suggesting that the extensively remodeled and functionally dynamic uterine vasculature is more vulnerable to particle exposure than vascular beds in the adult organism [162, 163]. For Ag NPs, the effects furthermore depended on particle size (only for 10 nm but not 100 nm Ag NPs) and surface modification (more substantial effect of citrate compared to PVP-coated Ag NPs) [162]. Using the less invasive inhalation route of exposure, TiO_2_ NPs have been shown to attenuate both the endothelium-dependent and -independent vessel dilation at the uterine-arteriolar level in both non-pregnant and pregnant rats [164, 165]. This was accompanied by significantly increased plasma levels of the pro-inflammatory factors IL-4 and IL-6 [165], indicating that systemic inflammation could have been implicated in the observed changes. Intratracheally instilled TiO_2_ NPs induced a systemic Th2 inflammatory response, which was mediated by group II innate lymphoid cells (ILC2) in the lungs. At the same time, endothelium-dependent dilation of the uterine radial arterioles was impaired [167]. IL-33 potently drives the production of Th2-associated cytokines. Treatment with an anti-IL-33 antibody prior to the TiO_2_ NP exposure attenuated the upregulation of circulating IL-33 levels and improved the endothelium-dependent dilation. Uterine microvascular dysfunction may, therefore, arise via activation of ILC2 cells in the lung and the subsequent systemic Th2-dependent inflammation. The study was performed in non-pregnant females, but the mechanisms ought to be also investigated during pregnancy. In another study, inhalation of TiO_2_ NPs during pregnancy was found to augment vasoconstriction of the uterine artery in response to Kisspeptin [166]. Kisspeptin is a potent vasoconstrictor and an essential reproductive hormone, which is secreted by the placenta during late pregnancy at 10,000-fold higher levels compared to the non-pregnant state (reviewed in [168]). It is, therefore, possible that TiO_2_ NP exposure perturbed the endocrine vascular axis via a kisspeptin-dependent mechanism [166]. Finally, a recent study investigated blood perfusion in placentas from pregnant rats inhaling TiO_2_ NPs. An endothelium-dependent increase in placental vascular resistance, measured as decreased outflow vein pressure, was observed. NO release plays a crucial role in maintaining the low basal tone of the materno-fetal circulation [169] and was therefore hypothesized to be implicated in the signaling causing the decrease in outflow pressure. Increased sensitivity to angiotensin II, a potent vasoconstrictor implicated in preeclampsia [170], was observed in both the placental and umbilical artery [96], also identifying the renin-angiotensin system as a perhaps crucial modulator of tissue tone.

Several studies observed abnormalities in placental development and function following exposure to NMs. Mice treated with 70 nm silica NPs (nSP70) i.v. (0.8 mg/mouse on GD 16–17) failed to form spiral artery canals, and blood flow in the fetal vascular sinuses decreased, perhaps due to abnormalities in structure and length of placental villi. The authors suggested that structural and functional changes to the placenta might be a result of the decreased levels of soluble Flt-1 [90], a potent anti-angiogenic factor involved in placental vascularization [171]. Orally administrated TiO_2_ NPs impaired placentation, possibly through the observed dysregulation of placental vascularization, proliferation and apoptosis [48]. Reductions were also observed for the expression of regulators for placental vascularization as well as the number of uterine natural killer (uNK) cells [48], a cell type that plays important roles in remodeling of spiral arteries, control of trophoblast invasion and placental development [172–174]. Injection of QDs (10 or 20 mg/kg body weight) on GD 13 was associated with placental vascular anomalies, as exposed rats displayed a reduced diameter of the labyrinth and basal zone and necrosis of invasive trophoblasts when examined on GD20 [97]. It is possible that the reduced placental size observed after QD treatment occurred due to the release of cadmium ions, as cadmium is a recognized placental toxicant [175]. A recent mouse study observed (non-particulate) cadmium to up-regulate several inflammatory cytokines in the placenta through the Akt signaling pathway [176], so the mechanisms of developmental toxicity of QD likely differ compared to that of other NMs. Vascular effects of NMs could involve the uptake of particles by extravillous trophoblasts resulting in impaired extravillous trophoblast invasion and vascular remodeling. Hence, a recent in vitro study found uptake of platinum NPs by autophagy to affect extravillous trophoblast functions in exposed cells [177]. Finally, there is also few hints that some NMs might interfere with placental barrier integrity and eventually transport. For instance, GO NPs induced a transient decrease in the integrity of BeWo trophoblast cells in vitro [103]. Blum et al., [83] suggested that developmental toxicity from CdO NP inhalation in mice might result from the accumulation of Cd ions and interference with transport in the placenta since a previous epidemiological study reported that placental Cd can alter the transport of zinc, vitamin B12 and other micronutrients [82]. However, conclusive evidence for NM effects on placental transfer in vivo is still lacking.In conclusion, it is becoming increasingly evident that exposure to NMs can interfere with vascular and placental development, structure and ultimately function, and, therefore, cause severe adverse effects on embryo-fetal development and offspring health. The underlying mechanisms, however, are still to be elucidated.

#### NM interference with extracellular vesicle signaling

Cells release extracellular vesicles (EVs) as part of their intercellular communication, with microvesicles (MVs) and exosomes as two main subgroups (reviewed in [178]). EVs contain various molecules such as lipids, nucleic acids and proteins, dependent on their donor cell type and physiological state, and the stimuli that mediated their formation and release [178]. The cargo is considered as information that EVs shuttle from donor to target cells, and even across internal barriers like the blood-brain-barrier [179–181]. Upon contact with the recipient cell, EVs have been shown to initiate different processes depending on their cellular origins, such as an immune response (exosomes originating from B lymphoblastoid or dendritic cells) [182, 183] or the transformation of healthy cells to tumor cells (MVs originating from MDAMB231 breast carcinoma and U87 glioma cells) [184].

During pregnancy, EVs are indicated to be involved in critical processes such as maternal-placental vascularization [185] and regulation of the maternal immune response [186]. However, when pregnant women suffer from certain diseases (e.g., preeclampsia and gestational diabetes mellitus), serum EV levels, composition, and function differ [187–192]. Even though it is still unclear, if the altered EV signaling is a contributing factor or only a symptom of a pregnancy complication, these studies indicate that EV levels and cargo reflect the physiological state of pregnancy.

Since NMs and EVs are of comparable size and circulate in similar compartments, interactions between them are considered possible, even though only few studies investigated this topic so far. In mice, intratracheal instillation of magnetic iron oxide NPs (MIONs with primary size ~ 43 nm) increased secretion of exosomes in the alveolar region, which contained increased levels of iron, albeit no MIONs could be imaged inside the vesicles by transmission electron microscopy [193, 194]. These exosomes were shown to initiate a systemic Th1-type immune response via direct and indirect T cell activation [193, 194]. In another study, subtoxic concentrations of ZnO (primary size ~ 10 nm) and TiO_2_ NPs (primary size of ~ 21 nm) did not significantly affect the characteristics of exosomes released by primary human peripheral blood mononuclear cells nor by monocyte-derived dendritic cells, such as size and number. Moreover, NPs were not found attached to the outside or inside of the exosomes [195]. In contrast, 20 nm sized gold NPs were taken up by human macrophages and found in released exosomes afterwards [196]. Interestingly, the only study investigating effects of NMs on placental-derived EVs demonstrated similar results. Human placental mesenchymal SCs released NP-loaded exosomes after exposure to hollow gold NPs (primary size 40 nm). These vesicles were further shown to exclusively migrate to the cell type of origin in vitro [197].

These studies indicate that the release of EVs and their characteristics can be altered by NM exposure, which could systemically affect other tissues in the body. In pregnancy, maternal exposure to NMs may lead to an EV-mediated modulation of cell communication between maternal, placental and fetal tissues. Considering that crucial processes during gestation are facilitated via EVs, disturbances in EV signaling could jeopardize pregnancy, which calls for more extensive investigation of potential influences of NMs on the EV system.

## Conclusions and future perspectives

Indirect pathways probably play a major role in NM-induced developmental toxicity. The placenta likely takes a central position in mediation hereof due to its location at the interface between mother and fetus and the many essential functions it undertakes during pregnancy. An increasing number of studies in experimental animals, in vitro and ex vivo models highlight how NM may exert effects indirectly via induction of maternal and placental oxidative stress and inflammation, activation of placental TLRs, impairment of placental growth, and secretion of placental hormones and vascular factors. Potentially, EVs could play a role in signaling between maternal and fetal organs. The impact of NMs on maternal and placental tissues can ultimately result in pregnancy complications and long-term effects on offspring health, even in the absence of or limited particle transfer across the placenta.

Our understanding of the involved mechanisms is, however, still scarce, and this emphasizes the need for more systematic studies. Comprehensive knowledge of the mechanisms underlying indirect toxicity increases the possibility of identifying triggers of effects and hence to categorize NMs based on shared properties of various materials. This will facilitate the risk assessment of human health effects, and furthermore, the application of a safe-by-design approach in the design of new materials [198]. An important issue is here, whether the effects of NP exposure depends on the existing state of inflammation in the mother, as e.g. asthma and obesity is associated with chronic low-grade inflammation [199]. To our knowledge, this aspect has only been marginally addressed. A study in pregnant mice indicated that LPS-induced intrauterine inflammation can increase the materno-fetal transfer of small AuNPs (3, 13 nm) after i.v. injection at GD 17 [135]. Identification of the key processes in NM induced developmental toxicity will further provide a basis for improvement of in vitro test systems, which are suitable for the screening of a broad range of NMs and allow for the identification of potential hazards to pregnancy. In addition, the application of advanced in vitro models would reduce the use of experimental animals [200].

Apart from identification of the mechanisms underlying developmental toxicity of NMs, the advancement of relevant in vitro systems will profit from the identification of the maternal and fetal organ systems that are most prone to disruption by NMs. To include pathways of indirect toxicity pathways, predictive developmental toxicity assessment of NMs requires the interconnection of multiple in vitro models such as the placenta, maternal tissues and the embryo, either directly in co-cultures [86, 87, 201], or indirectly via the transfer of conditioned media [88]. The potential of such approaches has been nicely exemplified in some recent studies on the indirect developmental toxicity of CoCr NPs, revealing DNA damage to occur across the placental barrier in neurons and astrocytes in the absence of NP transfer [86–88]. Recently, a novel microfluidic multitissue platform combining the embryonic SC test and liver microtissues for advanced embryotoxicity testing has been successfully implemented [202]. If the placental barrier could be integrated into such a platform, this might offer another auspicious approach for future developmental toxicity screening of NMs in a dynamic environment. However, the combination of different models often requires compromises and substantial modification of the cultivation conditions (e.g., culture medium, cultivation time), and thus, the predictive value of the newly developed multitissue models should be carefully validated. For instance, the use of serum-free media is required for cultivation of embryonic SCs or neural precursor cells to prevent their differentiation, but this might affect the protein corona of NMs, which could alter NM uptake and biological responses in the cells (reviewed in [203, 204]). Another challenge will be to distinguish between direct and indirect toxicity mechanisms for NMs that cross the placental barrier. Here, the comparison of different exposure conditions (e.g., direct versus indirect exposure to NMs as performed by Bhabra et al., (2009) [86]) or depletion of conditioned medium from NMs by centrifugation may allow to better understand the involvement of direct and indirect pathways. Finally, to identify novel mechanisms for indirect developmental toxicity mechanisms of NMs, unbiased omics approaches (e.g., transcriptomics, proteomics, secretomics or epigenomic profiling) should be explored to understand the array of molecular and functional changes that result from NM exposure.

## Data Availability

Not applicable.

## Abbreviations

Ag: Silver
BC: B cell
BL: Basal lamina
CB: Carbon black
CdO: Cadmium oxide
CdTe: Cadmium telluride
CeO2: Cerium dioxide
CoCr: Cobalt-chrome
CuO: Copper oxide
DC: Dendritic cell
DEP: Diesel exhaust particle
E: Endothelial
EVs: Extracellular vesicles
F: Fibroblasts
FC: Fetal capillary
GD: Gestational day
GI: Gastrointestinal
GO: Graphene oxide
HC: Hofbauer cell
hCG: Human chorionic gonadotropin
i.v.: intravenous
IGF: Insulin-like growth factor
ILC2: Innate lymphoid cell
LPS: Lipopolysaccharide
MIONs: Magnetic iron oxide NPs
MVs: Microvesicles
MWCNT: Multi-walled carbon nanotube
NAC: N-acetyl cysteine
Ni: Nickel
NM: Nanomaterial
NO: nitric oxide
NP: Nanoparticle
nSP70: 70 nm silica NPs
PLAP: Placental-type alkaline phosphatase
PlGF: Placental growth factor
PM: Particulate matter
QDs: Quantum dots
ROS: Reactive oxygen species
SC: Stem cell
sFlt-1: Soluble Flt-1
SiO2: Silica dioxide
ST: Syncytiotrophoblast
SWCNT: Single-walled carbon nanotube
TC: T cell
TiO2: Titanium dioxide
TLR: Toll-like receptor
UG: Uterine gland
uNK: Uterine natural killer
VEGF: Vascular endothelial growth factor
ZnO: Zinc oxide
